# Compositional profiling and sensory analysis of cauliflower by‐products‐enriched muffins

**DOI:** 10.1002/fsn3.3536

**Published:** 2023-07-20

**Authors:** Ammara Tukassar, Rizwan Shukat, Iahtisham‐Ul‐Haq, Masood Sadiq Butt, Gulzar Ahmad Nayik, Seema Ramniwas, Sami Al Obaid, Sulaiman Ali Alharbi, Mohammad Javed Ansari, Ioannis Konstantinos Karabagias, Nazmul Sarwar

**Affiliations:** ^1^ National Institute of Food Science and Technology University of Agriculture Faisalabad Pakistan; ^2^ Kauser Abdulla Malik School of Life Sciences Forman Christian College (A Chartered University) Lahore Pakistan; ^3^ Department of Food Science & Technology Government Degree College Shopian Jammu and Kashmir India; ^4^ University Centre for Research and Development Chandigarh University, Gharuan Mohali Punjab India; ^5^ Department of Botany and Microbiology, College of Science King Saud University Riyadh Saudi Arabia; ^6^ Department of Botany Hindu College Moradabad (Mahatma Jyotiba Phule Rohilkhand University Bareilly) Moradabad Uttar‐Pradesh India; ^7^ Department of Food Science and Technology, School of Agricultural Sciences University of Patras Agrinio Greece; ^8^ Department of Food Processing and Engineering Chattogram Veterinary and Animal Sciences University Chattogram Bangladesh

**Keywords:** antioxidant activity, by‐products, cauliflower, muffins, phytochemicals

## Abstract

Cauliflower (*Brassica oleracea* var*. botrytis*) by‐products (leaves, stems, stalks) (CBP) were successfully utilized in muffins as a model system and their feasibility of incorporation was investigated. CBP powder‐based muffin formulations were made by the progressive replacement of wheat flour (WF) with 10%, 20%, and 30% of CBP. The physicochemical, pasting properties, antioxidant potential, textural characteristics, and sensorial attributes were analyzed. Substitution of CBP significantly (*p* < .05) resulted in an upsurge in crude protein, crude fiber, minerals, total phenolics, and total flavonoid contents, as well as total antioxidant activity values of muffins. The pasting properties were influenced by monitoring an increase in peak, breakdown, final, and setback viscosities. Although the addition of an increasing amount of CBP improved the nutritional characteristics, however, the increased level of replacement (>10%) had significant adverse effects on baking and physical characteristics. The specific loaf volume of the developed muffins decreased the crumb color which became darker, and enriched muffins were hardened in texture. Furthermore, sensory evaluation confirmed the positive effects of CBP incorporation only up to 10%. Overall, present results highlighted that supplementation of wheat muffins with 10% CBP is a beneficial approach to enrich them with nutrients and intensify their antioxidant potential.

## Graphical abstract

Cauliflower by‐products (leaves, stems, stalks) were successfully utilized in muffins as a model system and their feasibility of incorporation was investigated. Cauliflower by‐products powder (CBP) based muffin formulations were made by the progressive replacement of wheat flour (WF) that resulted supplementation of wheat muffins with 10% CBP is a beneficial approach to enrich them with nutrients and intensify their antioxidant potential.

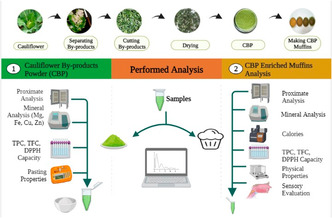

## 1 INTRODUCTION

Global food production is escalating day by day and consumers are gaining more awareness regarding health and well‐being. However, more than 1.3 billion tons of food produced annually ends up in the trash and results in an economic squander of $750 billion. Unfortunately, almost 815 million people in the world are suffering from hunger (Boliko, 2019). Consumption of green leafy vegetables is only 37% of the recommended daily intake. Agricultural waste is not only hindering food security, but also polluting the environment (Gebrechristos & Chen, 2018). For the achievement of a more sustainable world, reducing food waste should be one of the key objectives of ongoing research (Raak et al., 2017). The vegetable processing industry produces more than a million tons of vegetable by‐products as garbage, each year which is a potential source of bioactive ingredients (Galali et al., 2020; Rafiuddin et al., 2019, 2021). Studies have shown that there is a huge potential of utilizing by‐products from vegetable sources for developing food products (Amoah et al., 2023; San José et al., 2018; Tamasi et al., 2019). Cauliflower by‐products (CBP) are intended to hold a large share in this regard. Disposal of cauliflower waste (leaves, stems, stalks) contributes to about 60% of vegetable total weight and its highest waste index among all vegetables is a matter of concern (Ribeiro et al., 2015).

It has been underlined in various studies that inexpensive and intensively available underutilized by‐products of cauliflower are an exceptional source of proteins, vitamins, minerals, ascorbic acid, carotenoids, antioxidants, and dietary fibers (Montone et al., 2018; Munir et al., 2018; Stojceska et al., 2008). Previous studies have focused on the edible portion of cauliflower, but potential bioactive components of CBP have drawn recent scientific attention (Montone et al., 2018; Munir et al., 2018; Stojceska et al., 2008). These unexplored CBP have the potential to combat numerous chronic diseases. Antioxidants are considered to have the ability to scavenge free radicals and reduce oxidative stress (Awan et al., 2017). Bioactive polyphenols, sulforaphane, indoles, and peptides of CBP have promising antioxidant potential alongside their medicinal properties. Interestingly, these nutraceuticals offer chemotherapeutic properties due to their viable antioxidant, antihypertensive, anticancer, and anti‐inflammatory effects (Montone et al., 2018; Munir et al., 2018; Stojceska et al., 2008).

Cauliflower leaves have bioactive peptides found within native proteins that require to be released by enzymatic hydrolysis and bacterial fermentation during digestion. These bioactive peptides can improve the viability of human vascular endothelial cells by triggering the inhibition of intracellular xanthine oxidase activity and modulation of superoxide dismutase. Moreover, these peptides manifest antioxidant and ACE inhibitory properties. Hence, CBP could serve as supplementary protein sources (Caliceti et al., 2019). CBP flour contain substantial amounts of polyphenols including quercetin, kaempferol‐3‐O‐diglucoside‐7‐O‐glucoside, coumaric acid, caffeic acid, ferulic acid, sinapic acid, and total flavonoid glucosides (Gonzales et al., 2014). Furthermore, cauliflower and its by‐products contain glucosinolates in very high concentrations to 75,000 μg/g, and sinigrin is a vital glucosinolate that controls p53‐dependent pathway, thus, preventing abnormalities, necrosis, and further internal injury (Sanlier & Guler, 2018).

Baked and processed food products are often high in fat, which could result in lower diet quality and the progression of multiple ailments (Cummings et al., 2022). Abul‐Fadl (2012) incorporated white CBP flour as fat replacers in beef sausages and fat content was decreased in sausages from 28.40% to 35.54%. Ready‐to‐eat bakery products are consumed worldwide, and muffins are cherished worldwide among consumers of all age groups (Rabail et al., 2022). Muffins are made from wheat flour, sugar, fat, and eggs. Caloric values, physical characteristics, and physicochemical properties of processed muffins could be modified depending on the formulation, processing, and the used ingredients (Belorio & Gómez, 2020; Heo et al., 2019). Purposely, in this study, wheat flour was replaced with bioactive CBP flour to develop CBP‐enriched muffins. The primary scope of this study was to demonstrate the functional, nutritional, and sensorial attributes of CBP‐enriched muffins. Another complementary aim documented in the study was the introduction of an economical and nutrient‐dense ready‐to‐eat snack.

## 2 MATERIALS AND METHODS

### 2.1 Materials

The current research was conducted at the National Institute of Food Science and Technology (NIFSAT), University of Agriculture Faisalabad (UAF), Pakistan. For the investigation, commercial whole wheat flour (Sunridge, Karachi, Pakistan), vegetable oil (Dalda, Karachi, Pakistan), castor sugar (Sugarie, Karachi, Pakistan), low‐fat milk (Olper's, Karachi, Pakistan), baking powder (Rossmoor, Karachi, Pakistan), and whole eggs used in this study were purchased from the local markets in Faisalabad, Punjab, Pakistan. Cauliflower (*Brassica oleracea* var. *botrytis*) by‐products, including leaves, stems, and stalks were obtained from the vegetable processing industry in Pakistan.

### 2.2 Preparation of CBP powder

Cauliflower stems, stalks, and leaves were washed under running tap water and cut into small pieces. These were then blanched separately for 2–3 min and dried at room temperature for 1 h by spreading into a tray containing filter paper to rinse excess water. Afterward, all by‐products were individually dried in a hot air oven (Memmert GmbH + Co. KG, Buchenbach, Germany) at 60°C for 16 h till moisture remained at 6%–8%. Then, these by‐products were crushed to a fine powder with a laboratory grinder (Westpoint WF‐7701) and passed through 20 mesh sieves. Afterward, a homogenous mixture of all three by‐products was made by mixing them into equal proportions by weight and packed in airtight containers at ambient temperature till further use.

### 2.3 Muffins preparation

**FIGURE 1:**
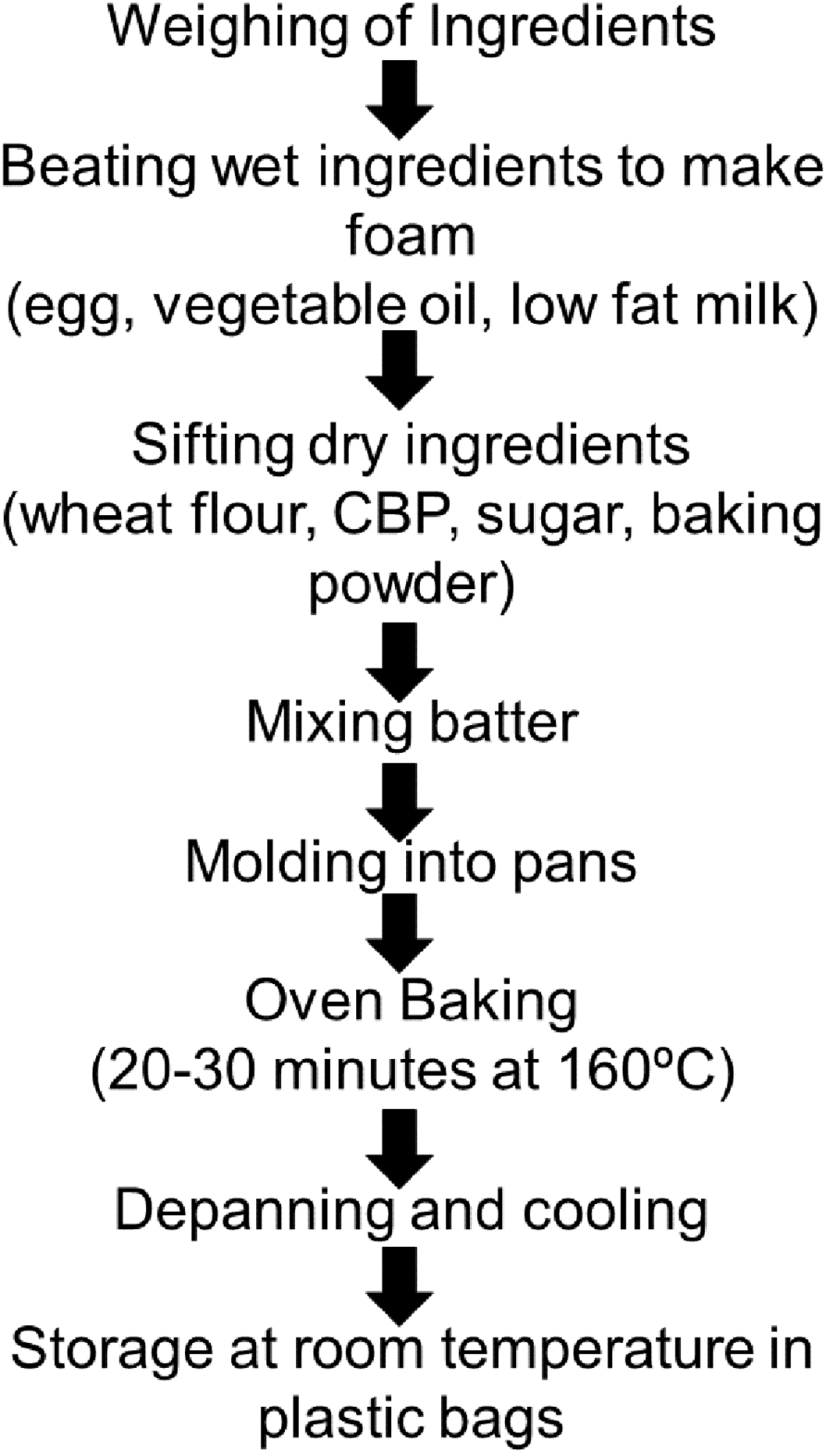
Flow chart showing the process of making Muffins.

The formulation presented in Table 1 was used for the muffin production. Muffins were prepared by substituting whole wheat flour with 0%, 10%, 20%, and 30% of CBP. The procedure of CBP‐enriched muffin preparation is demonstrated in Figure 1, indicating the main individual steps.

**TABLE 1:** Formulation of cauliflower by‐products (CBP)‐enriched muffins.

Ingredients (%)	Control	CBP10	CBP20	CBP30
Whole wheat flour	31.5	28.35	25.2	22.05
CBP powder	0	3.15	6.3	9.45
Low‐fat milk	6.6	6.6	6.6	6.6
Egg	24.6	24.6	24.6	24.6
Sugar	21.0	21.0	21.0	21.0
Canola oil	14.8	14.8	14.8	14.8
Baking powder	1.5	1.5	1.5	1.5

*Note*: Control: baked control muffins; CBP10: baked muffins with 10% CBP; CBP20: baked muffins with 20% CBP; CBP30: baked muffins with 30% CBP.

### 2.4 Nutritional composition

Proximate analysis parameters including moisture, fat, protein, fiber, ash, and carbohydrates were analyzed in wheat flour, CBP, and all muffin treatments, using standard methods (AACC, 2000). The moisture content was determined by the oven‐drying method. Approximately, 5 g of each sample was placed in a hot air oven for drying at 105°C for 24 h. Then, samples were put in a desiccator for 5 min to cool and were weighed again. The method no. 44‐15.02 described in AACC (2000) was followed to determine moisture content. Crude protein content was determined using the Kjeldahl apparatus (Inkjet, Germany) and method no. 46‐12 by AACC (2000) was used. First, 2 g of moisture‐free samples were digested with 25 mL of concentrated H_2_SO_4_ by using 5 g of digestion mixture (K_2_SO_4_:FeSO_4_:CuSO_4_ in 100:5:10 ratio) for 3–4 h until the color was light greenish or transparent. Next, the dilution of the digested material was done in a 250‐mL volumetric flask using distilled water until no residue was left. Then 10 mL of it were taken and 25 mL of alkali, that is, 40% NaOH were added to the distillation apparatus to get a nitrogen‐based sample in the form of NH_4_OH. Ammonia was liberated and collected in a beaker containing 10 mL of 4% boric acid solution using methyl red as an indicator. The resulting ammonium borate was used for nitrogen determination. Nitrogen was estimated by titrating distillate in the receiver flask against 0.1 N H_2_SO_4_ until the light pink color was obtained. Then crude protein was estimated by multiplying nitrogen % with factor 5.7 using the equations 1 and 2:

(1) $$\text{Protein}\;\left(\%\right)=\frac{\text{Volume of}\;0.1\;N\;H2\mathrm{SO}4\;\text{used}\;\left(\mathrm{mL}\right)\times \text{Volume of dilution}\left(250\mathrm{mL}\right)\times 0.0014}{\text{The volume of distillate taken}\times \text{weight of the sample}}\times 100$$

(2) $$\text{Crude protein}\;\left(\%\right)=\text{Nitrogen}\;\left(\%\right)\times 5.7$$

Furthermore, crude fat was determined using Soxhlet extractor (Thermo Fischer Scientific, USA), by following AACC (2000) method no. 30‐25. Approximately, 250 mL of *n*‐hexane, as a solvent, was added to 3 g of dried samples in a flask and connected to the apparatus. Then, tap water circulation was started in the condenser and heat was provided to receiving flask for 14 h. After 6–7 siphons, samples were dried in the hot air oven at 105°C for 1 h and then cooled in desiccators by sequential acid and alkali hydrolysis, and crude fat percentage was determined. Crude fiber content using the Fibertech apparatus (Labconco Corporation Kansas, USA) was estimated by digestion of fat‐free sample first in 1.25% H_2_SO_4_ and then in 1.25% NaOH solution by the AACC method no. 32‐10 (AACC, 2000). Additionally, after complete carbonization, samples were kept in a muffle furnace (Medsinglong, Guangzhou, China) at a temperature range of 550–600°C until grayish‐white ash was formed. Subsequently, these were placed in a desiccator for cooling and weighed again to quantify ash content. Finally, the following equation 3 was used to calculate carbohydrates:

(3) $$\text{Total carbohydrate}\;\left(\%\right)=100\%\left(\text{moisture}+\text{crude fiber}+\text{crude}\;\mathrm{fat}+\text{crude protein}+\mathrm{ash}\right)$$

Mineral elements including calcium (Ca), potassium (K), iron (Fe), copper (Cu), magnesium (Mg), and zinc (Zn) were determined by atomic absorption spectroscopy (Hitachi Polarized Zeeman AAS, Z‐8200, Japan) by using standard protocols (AACC, 2000). For this purpose, 3 g of samples were digested in a mixture of HNO_3_:HCLO_4_ in 7:3 over a hot plate and after digestion samples were diluted with distilled water to make a total volume of 250 mL. Samples were then run on atomic absorption spectrophotometer for determination of the respective minerals.

### 2.5 Pasting properties of muffin batters

Rheological properties of dough were determined according to general pasting method no. 76‐21.02 (AACC, 2000). Viscograms of flour samples were recorded by using a rapid visco analyzer (Super 4, Newport Scientific, Maryland, USA) and viscosity was expressed as rapid viscosity units (RVU).

### 2.6 Calories

The total calorific value of subsequent muffins was calculated using the Atwater factor by employing equation 4 as described by FAO (2003).

(4) $$\text{Energy}=\left(\text{Carbohydrate}\times 4\;\text{kcal}\right)+\left(\mathrm{Fat}\times 9\;\text{kcal}\right)+\left(\text{Protein}\times 4\;\text{kcal}\right)$$

### 2.7 Antioxidant assays and phytochemistry

Extracts of cauliflower waste (leaves, stems, and stalks), and prepared muffins, were made by following the protocol of Ansary et al. (2022). The total concentration of phenolic compounds was expressed as mg of GAE (gallic acid equivalents) per 100 g of dry weight. Total flavonoids were determined by following the Dowd method, absorbance was taken at 415 nm, and total flavonoid content was expressed as mg of catechin equivalents (mg CE/100 g DW) (Ahmed & Ali, 2013). In antioxidant activity analysis, the effect of the extracts on DPPH radical scavenging activity (%) (2,2‐diphenyl‐1‐picrylhydrazyl assay) was estimated, absorbance was taken at 515 nm and percentage inhibition activity was calculated (Widodo et al., 2019).

### 2.8 Physical characteristics of muffins

For the specific loaf volume of muffins, volume was determined using the rapeseed displacement method mentioned in AACC (2000) and weight was determined by using an electronic balance (Shimadzu UX4200H, Japan). The AACC method 74‐09.01 was adopted using a texture analyzer (TA. XT Plus Texture Analyzer, Stable Micro Systems, UK) equipped with a 20‐mm diameter and 40‐mm high –aluminum cylindrical probe. A double compression test was performed to determine the textural properties of muffins on samples that were cut into 2.5 cm cubes. Samples were compressed to 50% of their initial height. The computer was connected to a texture analyzer controlled system and texture expert program version 1.21 was used. The pretest speed was 5 mm/s, the test speed was 1 mm/s, the post‐test speed was 2 mm/s, and there was 30 s delay between two cycles. The parameters tested were hardness, cohesiveness, springiness, and chewiness.

The color parameters for CBP powder along with the crust colors of the muffins were determined by using a portable Hunter Lab colorimeter (Chroma Meter‐CR‐300, Konica Minolta Co. Ltd., Japan). Chromatic space of Cartesian coordinates L* a* b* (CIELAB) was used to evaluate the color. Colorimeter generally gives three values including the L* value (lightness) that varies from 0 to 100 (corresponding to absolute black and absolute white respectively), a* value that is represented by −*a* (green at one extremity) to +*a* (red at the other extremity), and b* value that varies from –*b* (i.e., blue) to +*b* (i.e., yellow). From a theoretical perspective, no extreme values exist for a* and b*, however, practically these are frequently numbered from −60 to +60. The total color difference (∆*E*) was calculated using equation 5 to quantify the overall color difference between a sample and the reference, which is in the control muffin sample (Barroca et al., 2017; Foucher et al., 2022).

(5) $$∆E=\sqrt{\left(L^{*}-L_{0}^{*}\right)+{\left(a^{*}-a_{0}^{*}\right)}^{2}+{\left(b^{*}-b_{0}^{*}\right)}^{2}}$$

where $L_{0}^{*}{,a}_{0}^{*}$, and $b_{0}^{*}$ are the color coordinates for the reference sample. A large total color difference corresponds to a greater color change from the reference sample. ∆*E* values in the range 0.0–2.0 correspond to unrecognizable differences, ∆*E* values within the interval 2.0–3.5 indicate differences possible to be recognized by an experienced observer, while ∆*E* values greater than 3.5 specify clear differences in the color of the samples under comparison (Guiné et al., 2018).

### 2.9 Sensory analysis

Sensory evaluation was conducted by 10 trained and 10 untrained panelists recruited (convenient consent‐based participant sampling) from the staff and students at the National Institute of Food Science and Technology, University of Agriculture Faisalabad. Muffins were evaluated based on their appearance, taste, texture, flavor, softness, palatability, color, and overall acceptability on a 9‐point hedonic scale (1 = dislike extremely, 5 = neither like nor a dislike, 9 = like extremely). The control muffin was presented simultaneously with the rest of the muffin samples and was evaluated in random order. Moreover, water was provided to panelists to minimize any residual effects before testing a new sample.

### 2.10 Statistical analysis

The experimental data were analyzed using “Statistics 8.1 Statistical Software.” To determine significant differences, ANOVA (one‐way analysis of variance) with Tukey's multiple comparison test was performed. The level of significance was set at *p* < .05.

## 3 RESULTS AND DISCUSSION

### 3.1 Compositional analysis of CBP

The proximate, nutritional, and physical properties of wheat flour (WF) and CBP powder are given in Table 2. Statistically significant (p < .05) differences were noted between the wheat flour and CBP powder. CBP powder had higher values of crude protein, crude fat, crude fiber, crude ash, Mg, Fe, Cu, Zn, total phenolic compounds, total flavonoid content, and DPPH inhibition activity as compared to wheat flour. On the other hand, moisture and carbohydrates content was higher in wheat flour. The results are in agreement with those reported by Munir et al. (2018), Chakraborty and Datta (2016), and Sharma and Prasad (2018). Results indicated that CBP are rich in nutritional value than wheat flour. The high moisture content can enhance spoilage, so vegetables are often dried to enhance their shelf life. Moreover, studies have demonstrated that CBP contain a complex mixture of bioactive compounds, thus, physiological activity could also be attributed to a synergistic action of distinct compounds according to van Breda and de Kok (2018). High fat represents high caloric density, so foods with reduced fat contents like CBP are of prime importance (Rios et al., 2014). A misconception is that protein needs are difficult to be met from vegetarian diets. Approximately 15%–25% of daily protein intake is recommended. A high intake of animal protein could lead to obesity in the future, so plant proteins are healthier for obese people (Marsh et al., 2013). Globally plants and animal foods contribute approximately 65% and 35% of protein, respectively. This shows that plants comprise a prominent place as protein sources (Wu, 2016). It has been reported that CBP comprised insoluble fibers including 17.32% cellulose, 9.12% hemicellulose, and 5.94% lignin (Khedkar et al., 2017). On a dried matter basis, cauliflower leaves contain 18.6% of total sugars, among which 3.6% are reducing sugars and 15.0% are nonreducing sugars (Wadhwa et al., 2006). Previous studies have also insisted that mineral contents in CBP are substantial to support bodily functions. Hence, CBP have been valorized as a potential source of antioxidants and possess significant free radical scavenging activity (Caliceti et al., 2019).

**TABLE 2:** Compositional profile of WF and CBP powder.

Parameters	Wheat flour (WF)	CBP powder
Moisture (%)	9.27 ± 0.07^a^	5.50 ± 0.04^b^
Crude protein (%)	10.66 ± 0.05^b^	22.80 ± 0.01^a^
Crude fat (%)	2.02 ± 0.01^b^	2.95 ± 0.02^a^
Crude fiber (%)	1.60 ± 0.01^b^	15.32 ± 0.07^a^
Crude ash (%)	0.30 ± 0.01^b^	5.81 ± 0.04^a^
Carbohydrates (%)	76.15 ± 0.06^a^	47.62 ± 0.03^b^
Mg (mg/kg)	182 ± 0.01^b^	363 ± 0.02^a^
Fe (mg/kg)	112.60 ± 0.05^b^	200.30 ± 0.09^a^
Cu (mg/kg)	1.70 ± 0.01^b^	8.30 ± 0.06^a^
Zn (mg/kg)	9.23 ± 0.04^b^	14.60 ± 0.07^a^
Total phenolic compounds (mg GAE/100 g)	116.50 ± 0.05^b^	537.40 ± 0.04^a^
Total flavonoid content (mg CE/100 g)	96.60 ± 0.04^b^	252.20 ± 0.02^a^
DPPH inhibition activity (%)	36.24 ± 0.02^b^	96.75 ± 0.07^a^

*Note*: Values containing different alphabetical letters in rows are statistically significant (*p* < .05). All data are represented as mean ± standard deviation.

Abbreviations: CBP, cauliflower by‐products; WF, Wheat flour.

### 3.2 Pasting properties

**FIGURE 2:**
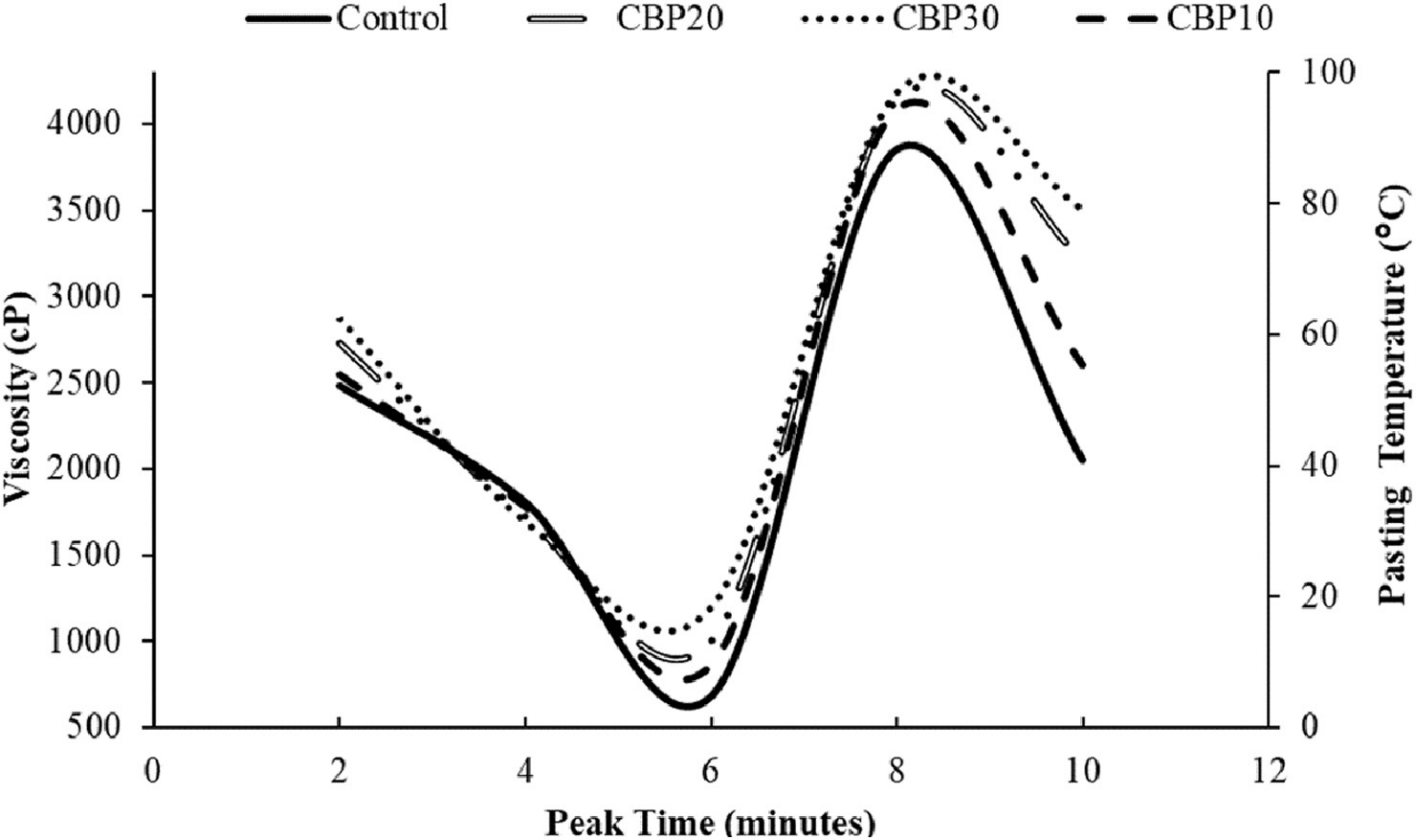
The pasting characteristics of composite batters. CBP10: baked muffins with 10% CBP; CBP20: baked muffins with 20% CBP; CBP30: baked muffins with 30% CBP.

The pasting properties of muffin batters, including peak viscosity, breakdown viscosity, final viscosity, and setback viscosity are shown in Table 3. Figure 2 represents a collective viscograph of all the treatments. Results depicted a significant (*p* < .05) increase in pasting properties with the incorporation of CBP (Wadhwa et al., 2006). It was reflected that the peak viscosity was 2483 ± 2.48 cP in the control treatment, which is the least value among all treatments, while it was highest, that is, 2875 ± 2.88 cP in CBP30. When starch–water‐based dough are heated, starch granules swell and rupture, leading to gelatinization. Granule swelling occurs because hydrogen bonds are formed between the hydroxyl groups of starch granule chains and water (Tarahi et al., 2022). Continued heating of these gelatinized granules led to an increased viscosity due to the leaching of amylose (Delatte et al., 2019). Moreover, it has been reported that the interaction between starch and nonstarch constituents, particularly proteins, could also lead to increased peak viscosity (Bravo‐Núñez et al., 2019). High peak viscosity also indicates high water binding capacity of CBP and more swelling power. In addition, high peak viscosity indicates further that starch granules in batters with more percentage of CBP would disintegrate more easily (Sopawong et al., 2022). This will result in fiber‐rich, denser muffins of low caloric value. Breakdown viscosity indicated resistance to heat, and it was at least 678 cP in control and a maximum of 1199 cP in CBP30. On the contrary, a lower trough viscosity of 1696 cP was exhibited by CBP30 as compared to other treatments. Setback viscosity was maximum for CBP30 and the higher the setback, the lower retrogradation or the gel‐forming ability of starch during cooling. Products with high setback viscosity have lower staling rates (Falade & Okafor, 2015). Final viscosity ranged from 3857 cP to 4183 cP and this marked increase could be attributed to the high realignment of amylose chains in starch after cooling (Flores‐Farías et al., 2000). The peak time indicates the ability of the mixture to reach peak viscosity and the maximum value for peak time was 6.04 min for control and the minimum value was obtained at 3.85 min for CBP30_._ Pasting temperature indicates the minimum temperature when viscosity starts to increase and it was highest for control muffins, while the lowest for CBP30 (Iwe et al., 2017). Another critical point to discuss is that the high starch content of the batters results in low pasting temperatures (Bravo‐Núñez et al., 2019). This could also be the underpinning reason that muffin batters with higher concentration of CBP have shorter cooking times, in contrast to the control muffins. Hence, pasting properties were affected after adding CBP, and this in turn affected the textural properties and digestibility of cooked muffins. It is well known that pasting properties have a direct link with proximate composition (Ocheme et al., 2018). CBP is high in overall insoluble fiber content and resistant starch therefore it results in thick muffin batters of high viscosity (Bakare et al., 2016; Kang et al., 2019).

**TABLE 3:** Pasting properties of CBP‐enriched muffin batter.

Muffins	Peak viscosity (cP)	Trough (cP)	Breakdown viscosity (cP)	Final viscosity (cP)	Setback viscosity (cP)	Peak time (min)	Pasting temperature (°C)
Control	2483 ± 2.48^d^	1805 ± 1.81^d^	678 ± 0.68^d^	3857 ± 3.86^d^	2052 ± 2.05^d^	6.05 ± 0.09^a^	88.1 ± 0.88^a^
CBP10	2549 ± 2.55^c^	1783 ± 1.78^c^	866 ± 0.87^c^	4089 ± 4.09^c^	2606 ± 2.61^c^	5.65 ± 0.09^b^	70.2 ± 0.7^b^
CBP20	2728 ± 2.73^b^	1725 ± 1.72^b^	1003 ± 1^b^	4135 ± 4.14^b^	3210 ± 3.21^b^	4.71 ± 0.07^c^	63.15 ± 0.63^c^
CBP30	2875 ± 2.88^a^	1696 ± 1.7^a^	1199 ± 1.2^a^	4183 ± 4.18^a^	3507 ± 3.51^a^	3.86 ± 0.06^d^	58.4 ± 0.58^d^

*Note*: CBP10: baked muffins with 10% CBP; CBP20: baked muffins with 20% CBP; CBP30: baked muffins with 30% CBP. Values containing different alphabetical letters in columns are statistically significant (*p* < .05). All data are represented as mean ± standard deviation.

### 3.3 Proximate and mineral composition of muffins

As shown in Table 4, moisture and carbohydrate content in muffins was significantly (p < .05) decreased, while a significant (p < .05) increase was observed for crude protein, crude fiber, and crude ash content, due to the addition of CBP powder. This might be explained by the richer protein, fiber, and ash content of CBP powder in contrast to wheat flour. The low moisture content is important for the enhanced shelf life of food products, as it reduces the proliferation of spoilage microorganisms. Control samples had a moisture content of 27.26%, while CBP30 had 23.91%. The reason for the decreased moisture in all muffins except control could be that some parts of the muffins had been replaced by the high fiber of CBP (Salazar et al., 2021; Singh et al., 2016). Fat content ranged from 6.44% in control and 6.82% in CBP30. Fat provides tenderness in baked products. CBP could serve as a supplementary plant protein source as CBP30 had the highest protein content of 9.7% in contrast to control muffins with only 7.53%. The amount of dietary fiber increased from 0.73 in control to 3.74% in CBP30. Ample amounts of dietary fiber in CBP powder incorporated muffins resulted in an increased density and reduced number of air pockets in muffins (Heo et al., 2019; Mirani & Goli, 2021). Ash content also followed the same increasing trend and was maximum in CBP30 which is 2.38%. On the other hand, carbohydrates decreased significantly (*p* < .05) from 56.24% to 53.46%, and this was a direct consequence of increasing CBP and decreasing WF concentration in muffins. Ribeiro et al. (2015) reported similar trends after CBP incorporation. Weight loss in all muffin treatments could be attributed to water evaporation mainly along with negligible liberation of oil (Shearer & Davies, 2005).

**TABLE 4:** The nutritional profile of cauliflower by‐products (CBP)‐enriched muffins.

Parameters	Control	CBP10	CBP20	CBP30
Moisture (%)	27.26 ± 0.27^a^	26.13 ± 0.26^b^	25.03 ± 0.25^c^	23.91 ± 0.24^d^
Fat (%)	6.44 ± 0.04^d^	6.55 ± 0.04^c^	6.69 ± 0.04^b^	6.82 ± 0.04^a^
Protein (%)	7.53 ± 0.08^d^	8.93 ± 0.09^c^	9.29 ± 0.09^b^	9.70 ± 0.10^a^
Fiber (%)	0.73 ± 0.01^d^	1.17 ± 0.02^c^	2.04 ± 0.03^b^	3.74 ± 0.06^a^
Ash (%)	1.82 ± 0.02^d^	2.01 ± 0.02^c^	2.17 ± 0.02^b^	2.38 ± 0.02^a^
Carbohydrates (%)	56.24 ± 0.06^a^	55.23 ± 0.06^b^	54.79 ± 0.05^c^	53.46 ± 0.05^d^
Calories (kcal)	134 ± 1.34^a^	126 ± 1.26^b^	114 ± 1.14^c^	106 ± 1.06^d^
Calcium (mg/100 g)	171.47 ± 1.71^d^	189.69 ± 1.9^c^	211.74 ± 2.12^b^	244.56 ± 2.45^a^
Potassium (mg/100 g)	108.19 ± 1.08^d^	119.51 ± 1.2^c^	132.58 ± 1.33^b^	167.76 ± 1.68^a^
Magnesium (mg/100 g)	51.35 ± 0.79^d^	53.94 ± 0.83^c^	57.52 ± 0.88^b^	61.19 ± 0.94^a^
Iron (mg/100 g)	1.66 ± 0.03^d^	2.66 ± 0.04^c^	4.64 ± 0.07^b^	6.56 ± 0.1^a^
Copper (mg/100 g)	0.26 ± 0.01^d^	0.68 ± 0.02^c^	1.49 ± 0.05^b^	1.99 ± 0.07^a^
Zinc (mg/100 g)	1.19 ± 0.02^d^	1.29 ± 0.02^c^	1.46 ± 0.02^b^	1.58 ± 0.02^a^

*Note*: CBP10: baked muffins with 10% CBP; CBP20: baked muffins with 20% CBP; CBP30: baked muffins with 30% CBP. Values containing different alphabets in rows are statistically significant (*p* < .05). All data are represented as mean ± standard deviation (*n* = 3).

Significant (*p* < .05) increases were obvious in the mineral contents of muffins, which were proportional to increases in the concentration of CBP added to muffin formulations (Table 4). Calcium increased from 171.47 mg in control to 244.56 mg in CBP30 samples. Potassium increased from 108.19 to 167.76 mg in CBP30, while magnesium increased from 51.35 to 61.19 mg. Iron content was uppermost in CBP30, with a value of 6.56 mg, and this is probably due to the presence of more CBP. Copper ranged from 0.26 mg in control to 1.99 mg in CBP30 samples, while zinc was 1.19 in control and 1.58 mg in CBP30 samples. Results indicated that CBP‐enriched muffins would contribute to meeting recommended daily intakes of minerals. These findings are consistent with those of Abul‐Fadl (2012) who reported that after the addition of CBP powder at different levels, mineral contents in developed products reached near the recommended dietary allowance (RDA) values. Wani et al. (2013) also reported that by supplementing WF with dried cauliflower leaves in noodles at different levels, improved the texture, taste, and overall acceptability of noodles.

### 3.4 Calorific value of muffins

By replacing WF with CBP, the energy values of muffins were significantly (*p* < .05) decreased. Calories in control were 134 kcal, while CBP30 had the lower calories equal to 106 kcal. The high fiber content in low‐caloric muffins with more CBP indicates their high amylose content as compared to control muffins. Amylose gelatinizes at elevated temperatures, so complete gelatinization of amylose is restricted during baking. This results in a more compact structure of muffins after cooling and the resistant starch contributes to a reduction in calories of muffins. Incorporated muffins, thus, will have higher satiety than control muffins due to the impact of CBP (Quilez et al., 2007).

### 3.5 Phytochemistry and antioxidant activity of muffins

**FIGURE 3:**
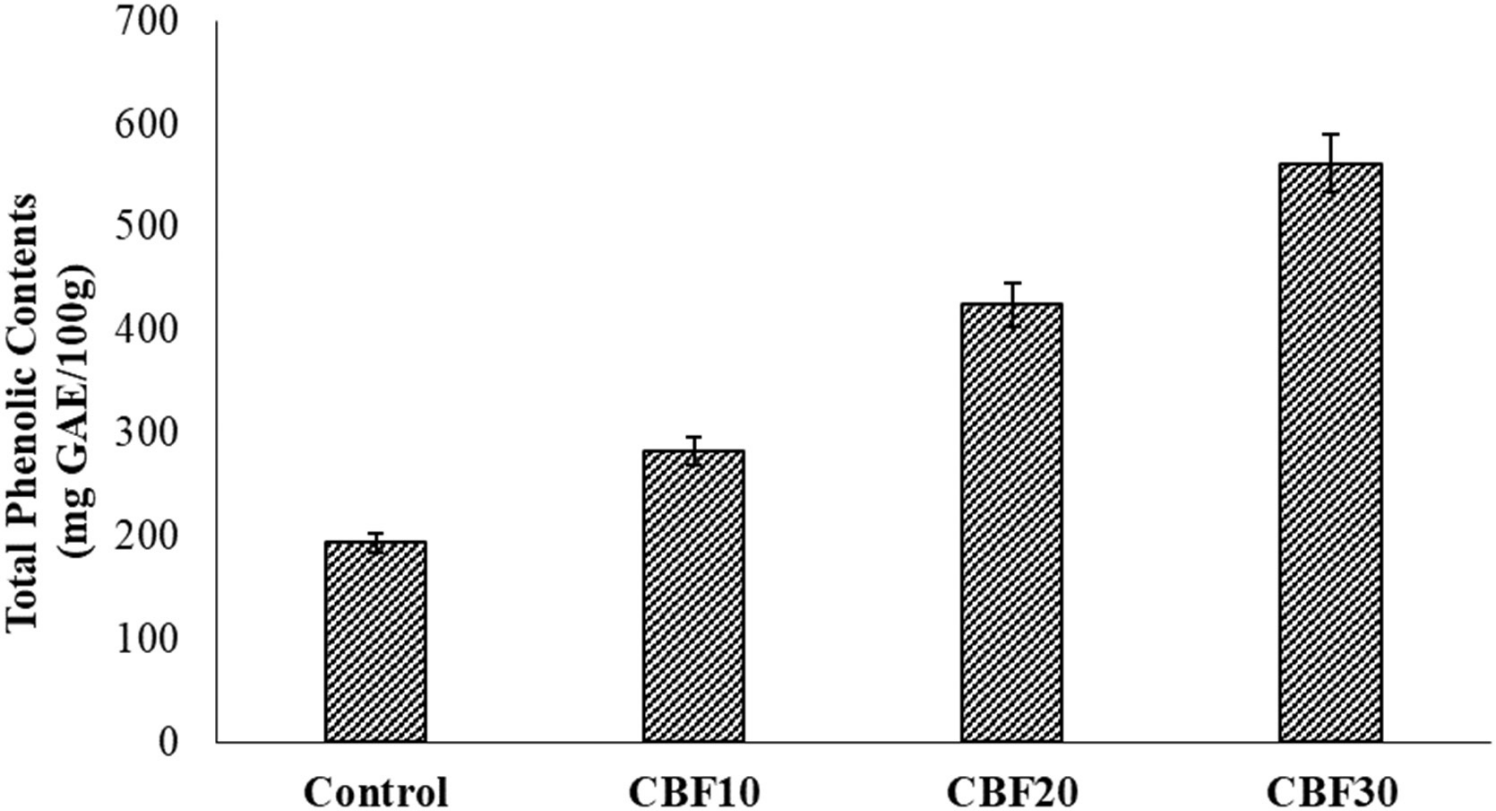
Effect of treatments on phytochemistry of cauliflower by‐products (CBP)‐enriched muffins; CBP10: baked muffins with 10% CBP; CBP20: baked muffins with 20% CBP; CBP30: baked muffins with 30% CBP.

**FIGURE 4:**
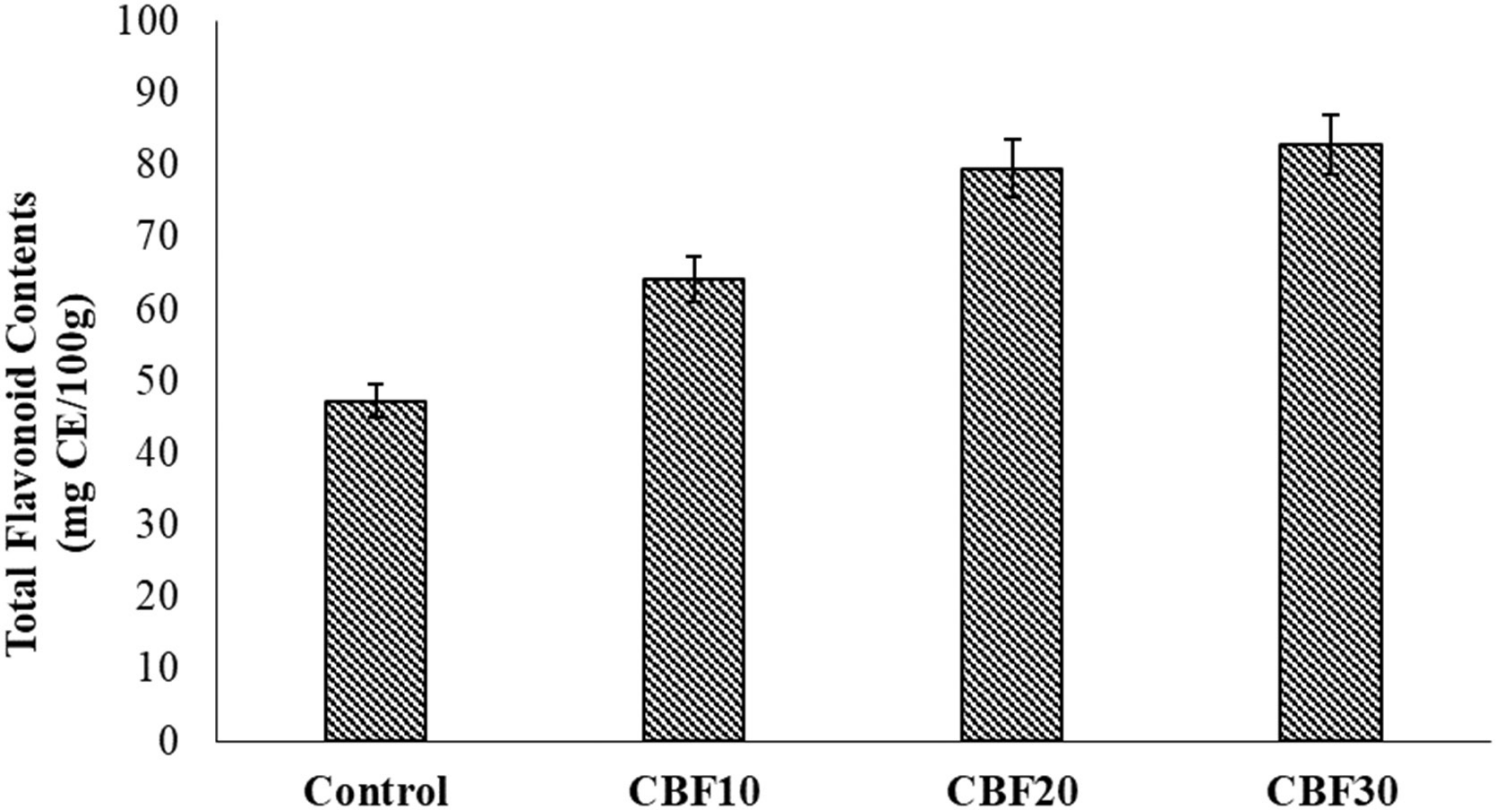
Effect of treatments on phytochemistry of cauliflower by‐products (CBP)‐enriched muffins; Control: baked control muffins; CBP10: baked muffins with 10% CBP; CBP20: baked muffins with 20% CBP; CBP30: baked muffins with 30% CBP.

**FIGURE 5:**
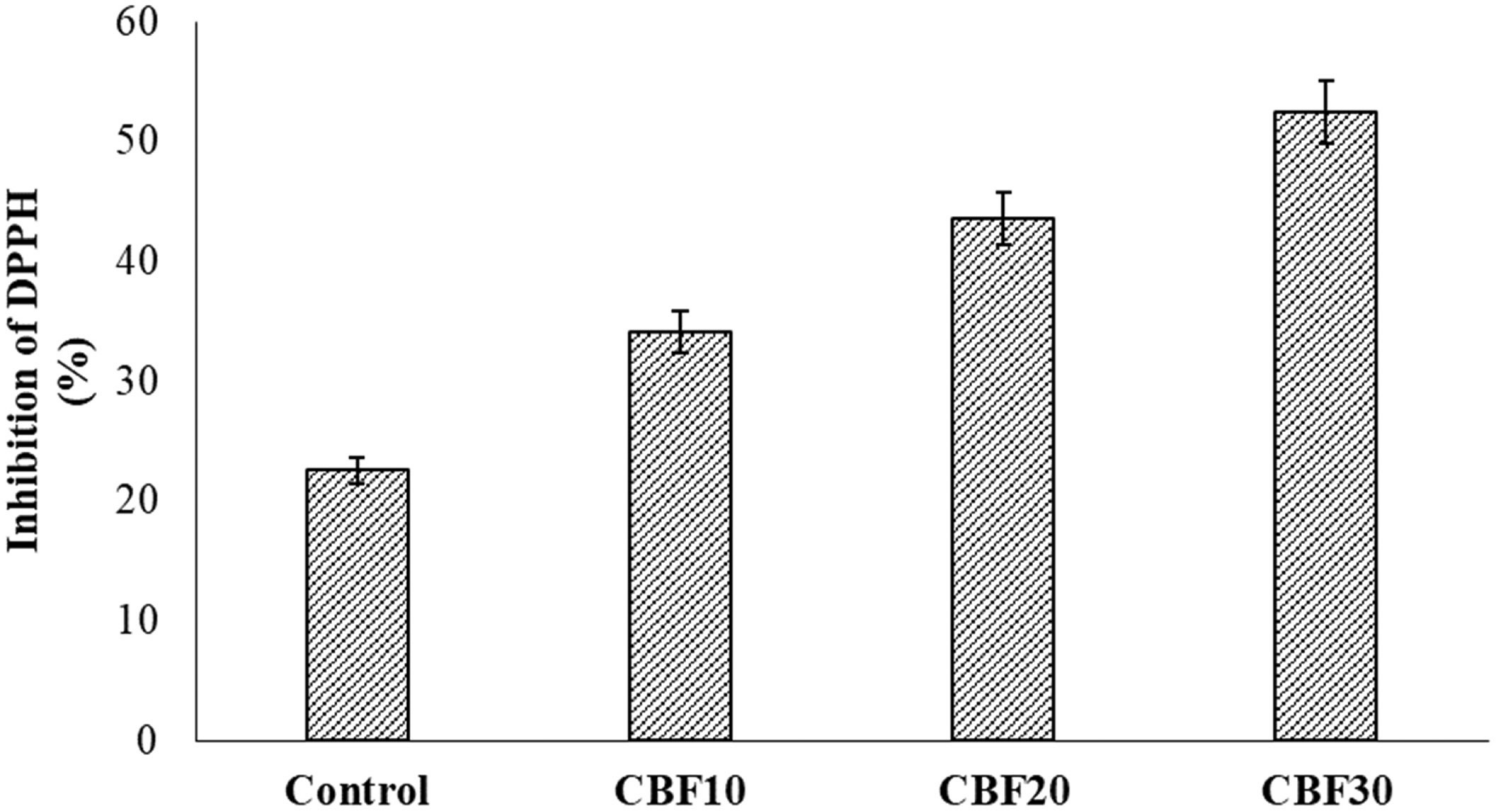
Effect of treatments on the antioxidant assay of cauliflower by‐products (CBP)‐enriched muffins; DPPH, 2,2‐diphenyl‐1‐picrylhydrazyl assay (%); Control: baked control muffins; CBP10: baked muffins with 10% CBP; CBP20: baked muffins with 20% CBP; CBP30: baked muffins with 30% CBP.

Muffins containing all levels of CBP had significantly (*p* < .05) higher total phenolic content, total flavonoid content, and antioxidant activity values than the control wheat muffins (Figures 3, 4, 5). The CBP10, CBP20, and CBP30 samples had total phenolic contents that were 282.40, 424.60, and 561.20 mg GAE/100 g, respectively. These values were two to three times higher than the control values of 193.10 mg GAE/100 g. Moreover, total flavonoid contents were 64.18, 79.53, and 82.78 mg CE/100 g in CBP10, CBP20, and CBP30, which were also two times higher compared to the control value of 47.24 mg CE/100 g. The DPPH inhibition activity was 22.53% in control, while the respective values of CBP10, CBP20, and CBP30 samples were 34.08%, 43.55%, and 52.41%, approximately 10%–30% higher than that of the control muffins. Results indicated that the enhanced concentration of polyphenols resulted in a high antioxidant capacity, showing the bioactive potential of CBP. Likewise, Aamer and Emara (2016) also reported the bioactive and antioxidant potential of phenolics in CBP. Generally, baking has comparatively less influence on nutrient loss than mixing time. The incorporation of air during prolonged mixing caused direct oxygenation as well. Some phenolic compounds are embedded in cell walls and for such compounds, baking helps in their better release, and thus, enhances their bioavailability (Bobinaitė et al., 2012; Mildner‐Szkudlarz et al., 2016).

### 3.6 Physical properties of muffins

Physical properties provide knowledge on how well the product is formed. Specific loaf volume is an indication of the degree of rising of the muffin dough. As shown in Table 5, the control treatment had the highest specific loaf volume of 2.05 cm^3^, followed by CBP10 with 1.98 cm^3^, then CBP20 with 1.91 cm^3^, and CBP30 with the lowest value of 1.82 cm^3^. Many factors like muffins' batter, whipping speed and time, baking temperatures, and overall mixture have an impact on the muffins' volume (Topkaya & Isik, 2019). In this study, it was noted that an increase in the concentration of CBP powder made the muffins' batter thicker and it was postulated that this thicker batter could be the reason for a decrease in volume values for muffins. Hence, it was clear that the addition of CBP was the main reason for the decreased trend of the specific loaf volume observed. The specific loaf volume of wheat muffins reported in the study of Kaur et al. (2020) was 2.165 cm^3^/g, which is close to the depicted value.

**TABLE 5:** Physical properties of cauliflower by‐products (CBP)‐enriched muffins.

Muffins	Specific loaf volume	Hardness (g)	Cohesiveness	Springiness (mm)	Chewiness (g/mm)	L*	a*	b*	∆*E* ^1^
Control	2.05 ± 0.02^a^	442.3 ± 0.44^d^	0.48 ± 0.02^a^	0.75 ± 0.02^a^	237.84 ± 0.24^d^	74 ± 0.74^a^	3.7 ± 0.19^a^	46 ± 0.46^a^	–
CBP10	1.98 ± 0.02^b^	623.4 ± 0.62^c^	0.45 ± 0.02^ab^	0.74 ± 0.02^ab^	259.65 ± 0.26^c^	62 ± 0.62^b^	2.5 ± 0.13^b^	32 ± 0.32^b^	19.45 ± 0.02^c^
CBP20	1.91 ± 0.02^c^	698.3 ± 0.7^b^	0.42 ± 0.02^bc^	0.71 ± 0.01^bc^	265.78 ± 0.27^b^	58 ± 0.58^c^	6.9 ± 0.36^c^	26 ± 0.26^c^	27.71 ± 0.02^b^
CBP30	1.82 ± 0.02^d^	717.2 ± 0.72^a^	0.39 ± 0.02^c^	0.69 ± 0.01^c^	270.60 ± 0.27^a^	55 ± 0.55^d^	8.4 ± 0.43^d^	21 ± 0.21^d^	33.65 ± 0.01^a^

*Note*: Control: baked control muffins; CBP10: baked muffins with 10% CBP; CBP20: baked muffins with 20% CBP; CBP30: baked muffins with 30% CBP. Values containing different alphabets in columns are statistically significant (*p* < .05) for specific loaf volume, hardness, chewiness, L*, a*, b*, and ∆*E*, while cohesiveness and springiness exhibited statistically insignificant values (*p* < .05). All data are represented as mean ± standard deviation (*n* = 3).

^1^ ∆*E* was calculated with control muffins as a reference.

Texture analysis directly influences consumer acceptability of the developed food products. The texture parameters of the muffins are presented in Table 5. The higher the CBP substitution levels, the greater (*p* < .05) the increase in the hardness value of muffins. Control had a hardness value of 442.3 g and the highest value was 717.2 g for CBP30. Higher hardness values for CBP muffins could also be associated with the gel‐forming properties of CBP (Hedayati et al., 2022). The reason for this could be attributed to the higher dietary fiber content of the CBP (Table 2). Dietary fiber can hold water, therefore, batter containing CBP was thicker, and this increased the hardness of the prepared muffins. This finding is supported by Kiran and Neetu (2017), who depicted that CBP‐incorporated muffins had a high hardness value of up to 693.8 g. The addition of CBP caused a minor decrease in cohesiveness from 0.48 in control to 0.39 in CBP30, springiness from 0.75 to 0.69 mm, and chewiness from 237.84 to 270.60 g/mm. However, the differences in values of cohesiveness and springiness among the muffin treatments were statistically insignificant (*p* > .05). Chewiness and hardness exhibited a similar increasing trend.

The crust color values of muffins in this study are given in Table 5. Statistically significant findings (*p* < .05) were obtained. Results indicated that Hunter L* value ranged from 55 to 74, the a* value ranged from 3.7 in control to −8.4 in CBP30, and the b* values were between 21 and 46. Likewise, Hunter colorimeter analysis was also performed for CBP powder, and results were recorded as L* = 38.29, a* = −1.57, and b* = 11.51. Not surprisingly, the negative value of a* indicated a distinguished greenness. *∆E* numerically showed the color difference between the control muffins and CBP muffin treatments. In this case, the *∆E* parameter was lowest in CBP10, that is, 19.45, while significantly higher values were reported for CBP20 and CBP30. The darker color of all the CBP muffins, in contrast to the control muffins, was a consequence of a higher substitution of CBP and heating effects. The color of muffins during baking is dependent on the Maillard reaction, sugar caramelization, besides the color of the used ingredients (Ureta et al., 2014).

### 3.7 Sensory evaluation

**FIGURE 6:**
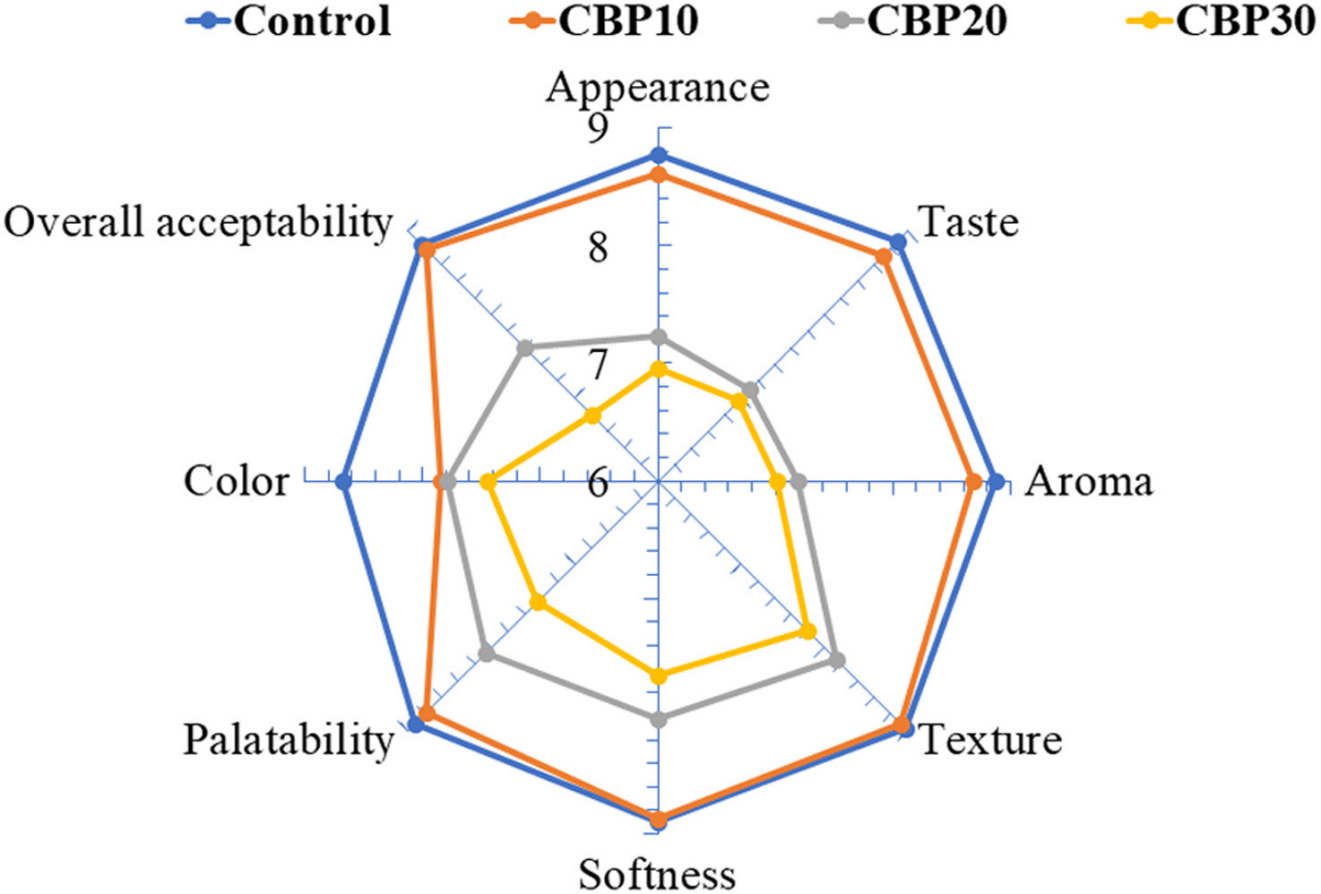
Sensory scores for CBP‐enriched muffins.

The sensory analysis results of the muffins are shown in Figure 6. A decrease in all attributes including appearance, taste, aroma, texture, softness, palatability, color, and overall acceptability was obtained for CBP20 and CBP30 muffins, but the results were not statistically significant (*p* < .05). The poor sensory scores of CBP20 and CBP30 could be due to the higher hardness values and increased viscosity of their batters due to CBP addition. Although muffins with 20% and 30% addition of CBP received lower sensory scores, results of control muffins and CBP10 muffins ranged between 8 and 9 for all attributes except color, which is a good indication. For color, panelists gave similar ratings, given that the visual observation of humans is not highly sensitive as a colorimeter. All CBP‐added muffins were perceived as slightly dark in the sensory evaluation room and were visually seen as having similar hues. In contrast to present findings, Wani et al. (2013) and Wani and Sood (2014) prepared noodles from CBP up to 10% and had similar eating qualities with those of the control. The overall sensory attributes of muffins enriched with CBP seem dependent on the amount of incorporation, which affects the total dietary fiber content of muffins and alters their consumer acceptance.

In terms of sensory evaluation scores, control muffins and CBP10 had almost similar scores in terms of appearance, taste, aroma, texture, softness, palatability, color, and overall acceptability. Based on the sensory evaluation results, it is not recommended to exceed 10% of CBP powder in muffins. Furthermore, more milk or any food essences could be added to muffins containing CBP powder to improve their chewiness, harness, and sensory and textural scores. However, future studies are needed in this regard.

## 4 CONCLUSION

In this study, CBP powder, which is recognized as a source of bioactive food components, was used in CBP‐enriched muffins production. This study promotes the preservation and utilization of cost‐effective cauliflower waste and probes its high antioxidant potential with special emphasis to develop nutrient‐rich muffins. The addition of cauliflower powder resulted in significant (*p* < .05) increase in protein, dietary fiber, Ca, K, Mg, Fe, Cu, Zn contents, total phenolic content, total flavonoid content, and total antioxidant activity values of muffins. Significant (*p* < .05) results were also obtained in color, texture, and other physical properties. A significant increase (*p* < .05) was observed in colorimeter values and hardness, while a significant (*p* < .05) decrease was noticed in specific loaf volume, chewiness, springiness, and cohesiveness scores with the addition of CBP. In terms of sensory evaluation scores, control and CBP10 had almost similar scores in terms of appearance, taste, aroma, texture, softness, palatability, and overall acceptability. Substantial contents of health‐promoting substances regard muffins as a novel confectionery product. Overall, muffins with 10% CBP powder were the most acceptable with good physicochemical, nutritional, and organoleptic attributes.

This study promotes the utilization of cost‐effective cauliflower waste and probes its high antioxidant potential with special emphasis to develop nutraceutical muffins. Results suggest that the phenolic profile of CBP‐enriched muffins has the potential to scavenge free radicals that may abridge chronic diseases as well as their underlying causes. Also, commercial products should be made by steeping the fortification of food products and endorsing the utilization of nutritious wastes. These products could be regarded as superfoods exhibiting high palatability, increased nutritional value, and bioavailability. This would also be beneficial in eradicating environmental pollution. The authors believe that CBP could be utilized in other foods also. Future studies relating to changes in shelf life, encapsulation of CBP powder, and bioavailability of these products are needed. CBP could potentially be incorporated as an ingredient to broaden its potential application in replenishing existing food products, resulting in economic benefits and this could minimize horticultural wastage as well. Moreover, future reformulation work could focus on optimizing the sensory quality of CBP‐fortified food products with the inclusion of up to 20% along with some flavorings.

## AUTHOR CONTRIBUTIONS

**Ammara Tukassar:** Data curation (equal); resources (equal); software (equal); writing – original draft (equal); writing – review and editing (equal). **Rizwan Shukat:** Investigation (equal); methodology (equal); validation (equal); writing – original draft (equal). **Iahtisham Ul Haq:** Conceptualization (equal); data curation (equal); software (equal). **Masood Sadiq Butt:** Formal analysis (equal); investigation (equal); methodology (equal); validation (equal). **Gulzar Ahmad Nayik:** Conceptualization (equal); funding acquisition (equal); resources (equal); writing – original draft (equal); writing – review and editing (equal). **Seema Ramniwas:** Formal analysis (equal); investigation (equal); methodology (equal); validation (equal). **Sami Al Obaid:** Funding acquisition (equal); methodology (equal); project administration (equal); resources (equal); supervision (equal); writing – original draft (equal). **Sulaiman Ali Alharbi:** Formal analysis (equal); funding acquisition (equal); investigation (equal); methodology (equal); supervision (equal). **Mohammad Javed Ansari:** Formal analysis (equal); funding acquisition (equal); software (equal); writing – original draft (equal). **Ioannis K. Karabagias:** Data curation (equal); resources (equal); validation (equal). **Nazmul Sarwar:** Conceptualization (equal); data curation (equal); validation (equal); writing – original draft (equal).

## CONFLICT OF INTEREST STATEMENT

The authors declare that they have no conflict of interest regarding the publication of this article.

## Data Availability

The datasets generated during and/or analyzed during this study are available from the corresponding author upon reasonable request.
